# Chronic kidney disease may evoke anxiety by altering CRH expression in the amygdala and tryptophan metabolism in rats

**DOI:** 10.1007/s00424-023-02884-y

**Published:** 2023-11-22

**Authors:** Katalin Eszter Ibos, Éva Bodnár, Hoa Dinh, Merse Kis, Fanni Márványkövi, Zsuzsanna Z. A. Kovács, Andrea Siska, Imre Földesi, Zsolt Galla, Péter Monostori, István Szatmári, Péter Simon, Márta Sárközy, Krisztina Csabafi

**Affiliations:** 1 Department of Pathophysiology, Albert Szent-Györgyi Medical School, University of Szeged, 1 Semmelweis utca, Szeged, H-6725 Hungary; 2 Department of Biochemistry, Bach Mai Hospital, 78 Giai Phong Street, Phuong Mai, Dong Da, Hanoi, 100000 Vietnam; 3 Department of Biochemistry and Interdisciplinary Centre of Excellence, Albert Szent-Györgyi Medical School, 9 Dóm tér, University of Szeged, Szeged, H-6720 Hungary; 4 Department of Laboratory Medicine, Albert Szent-Györgyi Medical School, University of Szeged, 6 Semmelweis utca, Szeged, H-6725 Hungary; 5 Metabolic and Newborn Screening Laboratory, Department of Pediatrics, Albert Szent-Györgyi Medical School, University of Szeged, 35-36 Temesvári körút, Szeged, H-6726 Hungary; 6 Institute of Pharmaceutical Chemistry and HUN-REN-SZTE Stereochemistry Research Group, University of Szeged, 6 Eötvös utca, Szeged, H-6720 Hungary

**Keywords:** Chronic kidney disease, Anxiety, Amygdala, Kynurenine, Corticotropine-releasing hormone, Uremic toxins

## Abstract

Chronic kidney disease (CKD) is associated with anxiety; however, its exact mechanism is not well understood. Therefore, the aim of the present study was to assess the effect of moderate CKD on anxiety in rats. 5/6 nephrectomy was performed in male Wistar rats. 7 weeks after, anxiety-like behavior was assessed by elevated plus maze (EPM), open field (OF), and marble burying (MB) tests. At weeks 8 and 9, urinalysis was performed, and blood and amygdala samples were collected, respectively. In the amygdala, the gene expression of *Avp* and the gene and protein expression of *Crh*, *Crhr1*, and *Crhr2* were analyzed. Furthermore, the plasma concentration of corticosterone, uremic toxins, and tryptophan metabolites was measured by UHPLC-MS/MS. Laboratory tests confirmed the development of CKD. In the CKD group, the closed arm time increased; the central time and the total number of entries decreased in the EPM. There was a reduction in rearing, central distance and time in the OF, and fewer interactions with marbles were detected during MB. CKD evoked an upregulation of gene expression of *Crh*, *Crhr1*, and *Crhr2*, but not *Avp*, in the amygdala. However, there was no alteration in protein expression. In the CKD group, plasma concentrations of p-cresyl-sulfate, indoxyl-sulfate, kynurenine, kynurenic acid, 3-hydroxykynurenine, anthranilic acid, xanthurenic acid, 5-hydroxyindoleacetic acid, picolinic acid, and quinolinic acid increased. However, the levels of tryptophan, tryptamine, 5-hydroxytryptophan, serotonin, and tyrosine decreased. In conclusion, moderate CKD evoked anxiety-like behavior that might be mediated by the accumulation of uremic toxins and metabolites of the kynurenine pathway, but the contribution of the amygdalar CRH system to the development of anxiety seems to be negligible at this stage.

**Supplementary Information:**

The online version contains supplementary material available at 10.1007/s00424-023-02884-y.

## Introduction

According to the current guideline of the Kidney Disease Improving Global Outcomes (KDIGO) initiative, chronic kidney disease (CKD) is defined as decreased kidney function shown by glomerular filtration rate (GFR) of less than 60 mL/min per 1.73 m^2^, or markers of kidney damage, or both, of at least 3-month duration. CKD is classified into several stages based on glomerular filtration rate and the extent of albuminuria [90].

The global burden of CKD is substantial, with more than 800 million people affected worldwide [46].

The progressive loss of kidney function results in a wide range of systemic complications, including anemia, electrolyte abnormalities, arterial hypertension, mineral bone disorder, and cardiovascular disease [2, 67]. Many patients also suffer from neurological sequelae, such as cognitive impairment, cerebrovascular diseases, and peripheral neuropathy [94], as well as mood disorders, i.e., anxiety and depression [27].

The prevalence of anxiety in end-stage kidney disease (ESKD) has been estimated to be around 12 to 52% [57], whereas in stages 3 and 4 of CKD 24.8% and 29.9%, respectively [49]. Multiple studies have identified an alarming link between anxiety and adverse clinical outcome in CKD [27]. Anxiety has been associated with more frequent hospitalizations, poor adherence to dialysis and an increased risk of all-cause and cardiovascular mortality in ESKD patients [72]. However, experimental data regarding anxiety in animal models of CKD have been scarce and controversial.

In recent years, a few studies have been conducted to characterize the effect of CKD on anxiety. Some results indicated an anxiogenic effect [41, 92], others have found CKD to be anxiolytic [16, 55, 80], and a very recent article reported that 2, 4, and 6 months after 5/6 nephrectomy in rats, no behavioral alterations were detected [65].

The background of anxiety in CKD has not been clearly described yet, though several hypotheses have emerged in the past decades.

Firstly, a hallmark of advancing CKD is the gradual retention of uremic toxins: organic solutes that accumulate in uremic patients and—in high concentrations—interact negatively with biological functions [81]. Apart from small, water-soluble compounds (e.g., urea, ammonia, asymmetric dimethyl-arginine), uremic toxins also include protein-bound compounds (e.g., indoxyl-sulfate, p-cresyl sulfate, kynurenine) and middle molecules of larger molecular weight (e.g., β2-microglobulin, atrial natriuretic peptide) [82]. Although protein-bound compounds had long been neglected due to their complicated kinetics and difficult removal by hemodialysis, recently, a growing number of publications have addressed the biological effects of these solutes [84].

Two compounds, indoxyl-sulfate (IS) and p-cresyl-sulfate (pCS), have been implicated in anxiety-like and depression-like behavior. In fact, in preclinical studies, the administration of IS in the drinking water of rodents has led to its accumulation in the CNS, modulating monoamine levels in several brain regions and causing anxiety-like and depression-like behavior, reduced locomotion and cognitive impairment [38, 76]. In patients with major depressive disorder, serum IS concentrations have positively correlated with total anxiety, psychic anxiety, and resting state functional connectivity of brain regions processing aversive stimuli [10].

Similarly to IS, pCS has also been associated with behavioral alterations in mice that underwent unilateral nephrectomy. After intraperitoneal pCS administration for 7 weeks, the animals developed anxiety-like and depressive-like behavior, as well as significant impairment in learning and spatial memory. These changes have been accompanied by a decrease in serum BDNF and serotonin concentrations and an increase in serum corticosterone levels. Moreover, pCS has inhibited neurotrophin-related intracellular signaling and induced oxidative stress and neuroinflammation [77].

Kynurenine (KYN) and other metabolites of the kynurenine pathway constitute another group of uremic toxins with immense biological significance. The KYN pathway is the main route for tryptophan (Trp) catabolism, controlled by the activity of 2 key enzymes: the hepatic tryptophan 2,3-dioxygenase (TDO) and indoleamine 2,3-dioxygenase (IDO) [59]. In CKD, multiple studies have reported an increase in KYN metabolites, some of which (e.g., 3-hydroxy-kynurenine (3-OH-KYN)) have been associated with increased oxidative stress, while others (e.g., kynurenic acid (KYNA)) have anti-inflammatory properties [3, 66, 88]. The main driver of KYN pathway activation in CKD is most likely chronic inflammation since inflammatory cytokines (such as IFN-γ, and TNF-α) activate IDO [95]. It is well established that CKD is associated with a pathological increase in the production of inflammatory cytokines, as well as reactive oxygen and nitrogen species (ROS, RNS) [35, 74]. A cohort study involving almost 4000 patients with CKD has also demonstrated a correlation between the plasma concentration of inflammatory biomarkers (IL-1β, IL-1 receptor antagonist, IL-6, TNF-α, C-reactive protein, and fibrinogen) and estimated GFR [28].

In addition, anxiety in CKD might be mediated by the dysregulation of the stress system. Corticotropin-releasing hormone (CRH) and arginine vasopressin (AVP) are central regulators of the stress system that govern the endocrine hypothalamic-pituitary-adrenal (HPA) axis. Wallace et al. have described an elevation of mean morning plasma cortisol concentration, an indicator of HPA axis activity, and an increase in 24-h total cortisol concentration with normal rhythmicity and resistance to dexamethasone suppression in ESKD patients [86]. Similarly, the progressive deterioration of kidney function from stage 1 to 4 has been associated with a continuous elevation of late-night salivary cortisol concentration. In some patients with stage 2–4 CKD, 1 mg of dexamethasone has not been sufficient to suppress cortisol secretion, pointing to the early disruption of feedback control [12]. CRH and AVP also by their expression in extrahypothalamic sites, such as in the amygdala-related circuitry [93], may mediate the stress-associated behavior. So far, it has not been explored in the literature if the expression of the CRH system and AVP is altered in any way in CKD. However, in ESKD patients, decreased functional connectivity has been detected in the amygdala by fMRI [14, 51] suggesting that an altered amygdala function might play a role in CKD-induced anxiety.

Considering the abovementioned data, the present study aimed to assess the effect of moderate CKD on anxiety in rats and to investigate if indeed uremic metabolites, altered HPA activity, and amygdalar CRH and AVP expression patterns might mediate the CKD-induced anxiety-like behavior. For that purpose, CKD was induced by 5/6 nephrectomy in adult male Wistar rats and confirmed by laboratory tests. At week 7, the animals underwent EPM, computerized OF, and marble burying (MB) tests to investigate anxiety-like behavior and locomotor activity. Two weeks after, the level of multiple plasma metabolites (including corticosterone, uremic toxins, compounds of tryptophan metabolism) was determined to create a complex metabolic profile of the 5/6 nephrectomy rat model. As HPA axis activation has been implicated in both CKD and anxiety, the expression of arginine vasopressin (*Avp*), corticotropin-releasing hormone (*Crh*), and CRH receptor 1 (*Crhr1*) and 2 (*Crhr2*) genes was determined in the amygdala, followed by analysis of protein expression.

## Materials and methods

### Animals

Adult male Wistar rats weighing 250–300 g were used in this study. Animals were housed in pairs in individually ventilated cages (Sealsafe IVC system, Italy) in a temperature-controlled room with a 12/12-h light/dark cycle. Standard rat chow and tap water were supplied ad libitum.

The animals were kept and handled during the experiments in accordance with the instructions of the University of Szeged Ethical Committee for the Protection of Animals in Research, which approved these experiments (XV./799/2019).

### Experimental setup

**Fig. 1:**
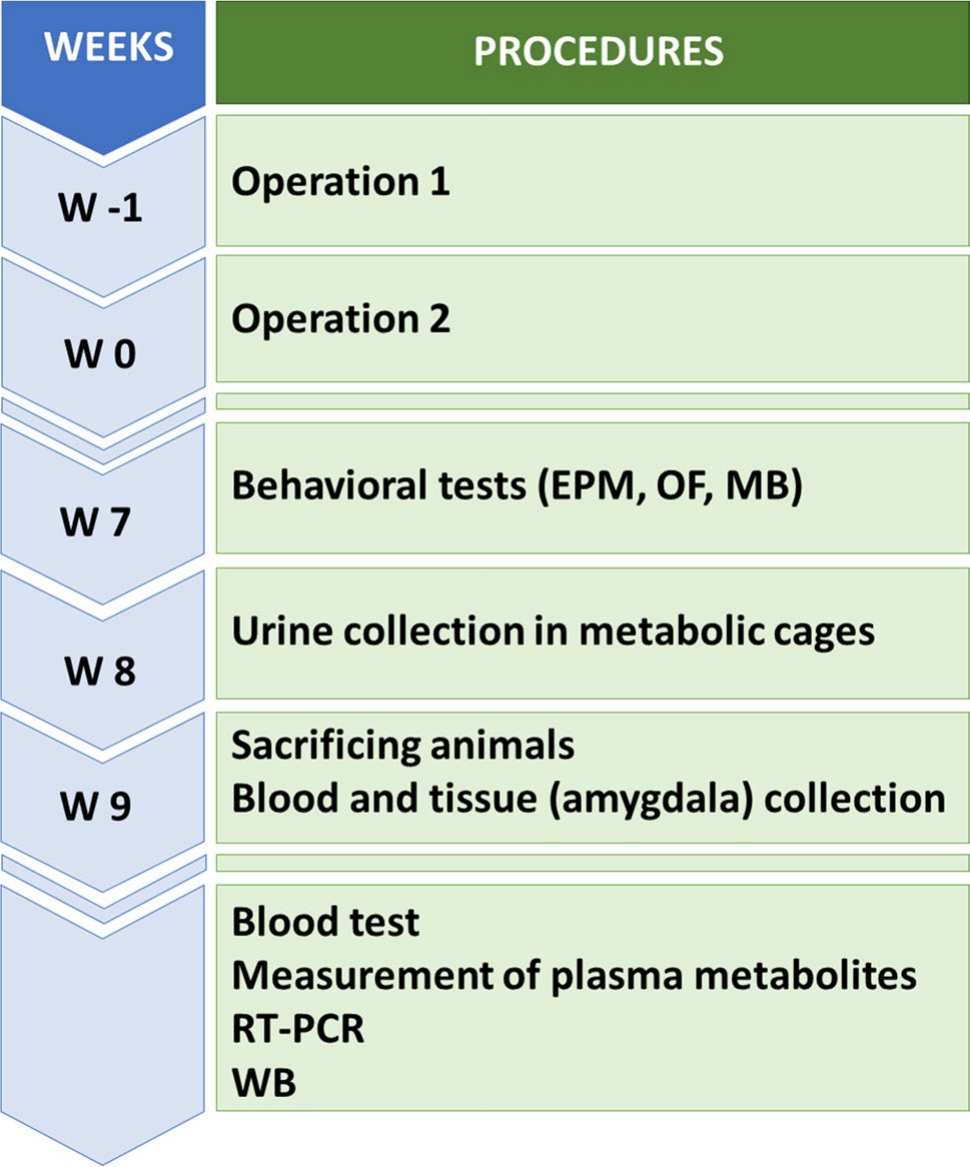
Experimental protocol. The second stage of 5/6 nephrectomy is considered “week 0”

CKD was induced by 5/6 nephrectomy. On the 7^th^ post-operative week, the animals underwent EPM, computerized OF, and MB tests to investigate anxiety-like behavior and locomotor activity. Urine, blood, and amygdala samples were collected on the 8^th^ and 9^th^ week, respectively, for further analysis. Figure 1 summarizes the timeline of procedures.

### Partial 5/6 nephrectomy

The animals underwent sham operation or 5/6 nephrectomy in two phases as described previously [68–70]. Anesthesia was induced by intraperitoneal (ip.) pentobarbital sodium (Euthasol; 40 mg/kg; Produlab Pharma b.v., Raamsdonksveer, The Netherlands). Laparotomy was performed to expose the left kidney followed by dissection of the adipose tissue and the renal capsule. Both poles were ligated approximately at the 1/3 position; then, the poles were excised.

One week after the first operation, a total right nephrectomy was performed following the same steps described above, in which the right kidney was dissected. The adrenal gland was gently freed and placed back into the abdominal cavity. Following the ligation of the ureter and renal blood vessels, the right kidney was removed. Control animals were subjected to a sham operation, during of which the renal capsule was removed. After both surgeries, the incision was closed with running sutures, and povidone iodide was applied to the surface of the skin. Subcutaneous (sc.) nalbuphine hydrochloride (0.3 mg/kg; Nalbuphine 10 mg/ml; TEVA, Debrecen, Hungary) was administered as a post-operative medication. Antibiotics (Enroxil, 75 mg; Krka, Slovenia) and analgesics (10 mg/L of nalbuphine hydrochloride, Nalbuphine; TEVA) were administered in tap water for 2 days after both surgeries.

### Blood test and urinalysis

#### Urine creatinine and total protein levels

At week 8, a subgroup of animals was placed in metabolic cages (Tecniplast, Italy) to collect urine for 24 h. To verify the development of CKD, urine creatinine and urine protein levels were measured by standard laboratory methods as described previously [43, 68]. Moreover, the urine protein/creatinine ratio was calculated to assess proteinuria.

#### Serum carbamide and creatinine levels

Blood was collected from the thoracic aorta at week 9 to measure serum carbamide (urea) and creatinine levels to verify the development of CKD. Urea and creatinine levels in serum were quantified by the kinetic UV method using urease and glutamate dehydrogenase enzymes and the Jaffe method, respectively. The reagents and the platform analyzers were from Roche Diagnostics (Mannheim, Germany) [68, 69].

#### Calculation of estimated glomerular filtration rate (eGFR)

The calculation of eGFR at week 9 was performed using serum creatinine and urea concentrations, as well as body weight. The following formulae by Besseling et al. were used [5]:

$$\textrm{Plasma}\ \textrm{creatinine}<\frac{52\upmu \textrm{mol}}{L}: eGFR=880\times {W}^{0.695}\times {C}^{-0.660}\times {U}^{-0.391}$$

$$\textrm{Plasma}\ \textrm{creatinine}>52\ \upmu \textrm{mol}/L: eGFR=5862\times {W}^{0.695}\times {C}^{-1.150}\times {U}^{-0.391}$$

In the formulae, *W* is body weight (g), *C* is serum creatinine concentration (μmol/L), and *U* is serum urea concentration (mmol/L).

#### Serum ion levels

Serum sodium, potassium, calcium, magnesium, phosphate, and chloride levels were determined by indirect potentiometry using ion-selective electrodes at week 9. All reagents and instruments were from Roche Diagnostics (Mannheim, Germany) [68].

### Synthesis of p-cresyl-sulfate (pCS)

The applied p-cresyl-sulfate (pCS) was synthesized based on the literature method [54] implementing different optimizations.

To the stirred solution of p-cresol (1.0 g; 9.26 mmol) in 10 mL cooled pyridine 1.3 g (11.16 mmol) of chlorosulfonic acid was added dropwise while maintaining the temperature. Stirring it for 8 h, aqueous KOH (50 %) was added in excess to basify the solution. The basic solution was stirred at room temperature for an additional 1 h; then, the formed salts were filtered and washed with diethyl ether. From the crystals, warm ethanol was used to extract the cresyl-sulfate salt; then, after concentration, the residue was crystallized with cool ethanol and recrystallized from ethanol: diisopropyl ether (1:1). Yield: 1.47 g (70%), white powder, m. p.: 188–190°C.

The structure and the purity of the synthesized pCS were supported by its ^1^H-NMR (see Figure S1 in the Supplement [https://data.mendeley.com/datasets/gg2nrgfnzb/draft?a=91893794-72f9-4804-8f73-a80c5a429c90]).

### Measurement of plasma metabolites

Plasma samples were collected at week 9, prepared [24], and measured [23] according to previously published methodologies using ultra-high performance liquid chromatography-tandem mass spectrometry (UHPLC-MS/MS). MRM transition of pCS was 186.9/107.0 using −50 V as declustering potential and −26 V as collision energy, retention time: 12.50 min. MRM transition of IS was 211.9/131.9 using −50 V as declustering potential and −25 V as collision energy, retention time: 11.48 min. MRM transition of Picolinic acid was 124.0/106.0 using 75 V as declustering potential and 13 V as collision energy, retention time: 1.21 min. MRM transition of Corticosterone was 347.2/121.1 using 50 V as declustering potential and 30 V as collision energy, retention time: 13.14 min.

### Behavioral tests

#### Elevated plus maze

Anxiety-like behavior was assessed using the EPM at week 7. The EPM apparatus is a plus-shaped platform 50 cm above the ground. The maze consists of four arms (50 cm x 10 cm each): two opposing open arms and two closed arms enclosed by a 10 cm high wall. The test is based on two conflicting motivations of rodents: to avoid open, brightly lit spaces and to explore novel environments. The avoidance of open arms reflects anxiety-like behavior [85]. The experiments were conducted between 8 a.m. and 10 a.m. The apparatus was cleaned with 96% ethyl alcohol after each session. Rats were placed in the maze facing one of the open arms; then, their behavior was recorded by a camera suspended above the maze for 5 min. The time spent in each arm, as well as the number of entries per arm were registered by an observer blind to the experimental groups.

#### Open field

The novelty-induced locomotor activity of rats was assessed using the Conducta 1.0 System (Experimetria Ltd., Hungary) 7 weeks after nephrectomy. The system consists of five black plastic OF arenas (inside dimensions: 48×48 cm, height: 40 cm) with 5 horizontal rows of infrared diodes on the walls to register both horizontal and vertical locomotion. The center of each box is illuminated by a LED lightbulb (230 lumens) from above the box. The central zone of the arena is defined as a 24×24 cm area in the center of the box. The OF experiments were conducted between 8 a.m. and 10 a.m. 7 weeks after nephrectomy. The rats were placed in the center of the box and their behavior was recorded by the Conducta computer program for 5 min. Six behavioral parameters were measured during the experiment: total time and total distance of ambulation, immobility time, number of rearings (vertical locomotion), time spent in the central zone (central area of 24×24 cm), and distance traveled in the central zone. The apparatus was cleaned with 96% ethyl alcohol after each session.

#### Marble burying

MB is a regularly used paradigm for the assessment of anxiety-like and compulsive-like behavior [9] that was performed 7 weeks after nephrectomy. Our protocol was based on the method described by Schneider and Popik [71]. The animals were removed from their plexiglass home cages (420×275×180 mm) and temporarily moved into another cage before the experiment. Meanwhile, the home cage was prepared for the experiment by increasing the depth of bedding material to 5 cm and arranging 9 glass marbles of 2.5 cm diameter in 3 rows along the shorter wall of the cage. The experiment was conducted for 10 min and recorded by a video camera above the cage. After the session, the animal was removed from the cage and the number of buried marbles (>50% marble covered by bedding material) was counted. The marbles were cleaned with 96% ethyl alcohol after each session. After the experiment, the count and duration of two types of goal-oriented interactions with marbles (burying of marbles and moving marbles without burying them) were assessed by an observer blind to the experimental group.

### Gene expression

#### Sample preparation

Following decapitation, the brains of the rats were gently removed and immediately dissected with a pre-cooled adult rat brain matrix (Ted Pella Inc., Redding, CA, USA) at week 9. Next, brains were manually sliced with pre-cooled razor blades in coronal sections (1 mm slots), after of which a 2-mm tissue puncher was used to obtain samples of the amygdala, according to the brain atlas of Paxinos [63]. The tissue samples were stored in 1 mL of TRIzol (UD-GenoMed, Hungary) in Eppendorf tubes and kept in a freezer at -80°C.

#### RNA purification

The tissue samples underwent ultrasonic homogenization (Branson Sonifier 250, Emerson, USA); then, 200 μL of chloroform was added to each sample. Following 10 min of incubation at room temperature, the samples were centrifuged for 15 min at 13000 g at 4°C (Heraeus Fresco 17, Thermo Fisher Scientific, USA). Approximately 500 μL of supernatant was collected from each tube and transferred to new Eppendorf tubes containing 600 μL of cold 96% alcohol that was stored overnight at -20°C. On the following day, GeneJET RNA Purification Kit (Thermo Fisher Scientific, USA) was used according to the manufacturer’s instructions. The concentration of the purified samples was calculated based on the average of three measurements with a spectrophotometer (NanoDrop One^C^, Thermo Fisher Scientific, USA).

#### cDNA synthesis

A volume containing 300 ng of RNA was obtained from each sample for cDNA synthesis. The first strand cDNA was synthesized using the Maxima First Strand cDNA Synthesis Kit (Thermo Fisher Scientific, USA) according to the manufacturer’s instructions.

#### RT-qPCR

The qPCR reaction mix was prepared using the Luminaris Color HiGreen Low ROX qPCR Master Mix (Thermo Fisher Scientific, USA) according to the manufacturer’s instructions. A total volume of 10 μL of reaction mix was prepared, containing 5 μL of Master Mix, 0.3 μL of forward primer, 0.3 μL of reverse primer, 1.67 μL of cDNA, and 2.73 μL of nuclease-free water. The custom primers corresponding to the *Avp*, *Crh*, *Crhr1*, and *Crhr2* genes are shown in Table 1. The mix was placed in a thermal cycler (C1000 Touch Thermal Cycler, BioRad) which was programmed according to the cycling protocol in Table 2. The expression of each gene relative to GAPDH was determined using the ΔΔCT method.

**Table 1:** Custom primers

	Forward	Reverse
*Avp*	5′-CTG ACA TGG AGC TGA GAC AGT-3′	5′-CGC AGC TCT CGT CGC T-3′
*Crh*	5′-TGG TGT GGA GAA ACT CAG AGC-3′	5′-CAT GTT AGG GGC GCT CTC TTC-3′
*Crhr1*	5′-CGA AGA GAA GAA GAG CAA AGT ACA C-3′	5′-GCG TAG GAT GAA AGC CGA GA-3′
*Crhr2*	5′-CCC GAA GGT CCC TAC TCC TA-3′	5′-CTG CTT GTC ATC CAA AAT GGG T-3′
*Gapdh*	5′-CGG CCA AAT CTG AGG CAA GA -3′	5′TTT TGT GAT GCG TGT GTA GCG-3′

**Table 2:** qPCR cycling protocol

Step	Temperature °C	Time	Number of cycles
UDG pre-treatment	50	2 min	1
Initial denaturation	95	10 min	1
Denaturation	95	15 s	40
Annealing	60	30 s	
Extension	72	30 s	

### Western blot

As described previously, a standard Western blot technique was used [22, 45] to investigate the expression at the protein level of CRH (21 kDa) with β-actin (45 kDa); CRHR1 (50 kDa), and CRHR2 (47 kDa) with GAPDH (37 kDa) loading background. Amygdala samples (*n*=26) were homogenized with an ultrasonicator (UP100H, Hielscher Ultrasonics GmbH, Germany) in Radio-Immunoprecipitation Assay (RIPA) buffer (50 mM Tris–HCl (pH 8.0), 150 mM NaCl, 0.5% sodium deoxycholate, 5 mM ethylenediamine tetra-acetic acid (EDTA), 0.1% sodium dodecyl sulfate, 1% NP-40; Cell Signaling Technology Inc., USA) supplemented with phenylmethanesulfonyl fluoride (PMSF; Sigma-Aldrich, USA), sodium orthovanadate (Na_3_VO_4_ ; Sigma-Aldrich, USA), and sodium fluoride (NaF; Sigma-Aldrich, USA). The crude homogenates were centrifuged at 15,000×g for 30 min at 4 °C. After quantifying the supernatants’ protein concentrations using the BCA Protein Assay Kit (Pierce Thermo Fisher Scientific Inc., USA), 50 μg of reduced and denaturized protein was loaded. Then sodium dodecyl-sulfate polyacrylamide gel electrophoresis (SDS-PAGE, 50 V, 5 h) was performed on 12% gel for CRH and 10% gel for CRHR1 and CRHR2, followed by the transfer of proteins onto a nitrocellulose membrane (10% methanol, 35 V, 2 h). The efficacy of transfer was checked using Ponceau staining. The membranes were cut vertically and horizontally into parts corresponding to the molecular weights of each protein. Membranes were blocked for 1 h in 5% (w/v) bovine serum albumin (BSA, Sigma-Aldrich, USA) supplemented with Na_3_VO_4_ and NaF, and were incubated with primary antibodies in the concentrations of 1:500 against CRH (# BS-0382R, ThermoFisher Scientific Inc., USA) and CRHR1 (#SAB4500465, Sigma-Aldrich, USA); 1:1000 against CRHR2 (#SAB4500466, Sigma-Aldrich, USA) and β-actin (#4970S, Cell Signaling Technology Inc., USA) and 1:5000 against GAPDH (#2118, Cell Signaling Technology Inc., USA) overnight at 4 °C in 5% BSA. Then the membranes were incubated with IRDye 800CW Goat Anti-Rabbit (LI-COR Biosciences, Lincoln, NE, USA, in the concentration of 1:20000) for 1 h at room temperature in 5% BSA to detect proteins with similar molecular weight on the same membrane where it is applicable. Fluorescent signals were detected by the Odyssey CLx machine (LI-COR Biosciences, Lincoln, NE, USA), and digital images were analyzed and evaluated by densitometry with Quantity One Software (Bio-Rad Laboratories Inc., USA). The full-length Ponceau-stained membranes and corresponding Western blot images are presented in Supplementary Figures S2-4 (https://data.mendeley.com/datasets/gg2nrgfnzb/draft?a=91893794-72f9-4804-8f73-a80c5a429c90).

### Statistical analysis

Data are presented as means ± SD. All statistical analyses were performed using GraphPad Prism 9.1.2. Graphs and figures were created using GraphPad Prism 9.1.2 and Microsoft PowerPoint 2016. Unpaired *t*-test was used for the statistical analyses of the blood test and urinalysis results. Welch’s unpaired *t*-test was employed for the evaluation of OF test results. The EPM, MB, and PCR findings were assessed using Mann-Whitney’s test. A probability level of 0.05 or less was accepted as indicating a statistically significant difference.

## Results

### Blood test and urinalysis confirmed the development of CKD

The results of the blood test and urinalysis are summarized in Table 3. Compared to the sham-operated group, a significant increase in serum carbamide (*p*<0.0001) and creatinine (*p*<0.0001) concentrations was detected 9 weeks after 2/3 nephrectomy. Likewise, serum potassium (*p*=0.0329), calcium (*p*=0.0013), magnesium (*p*=0.0083), and phosphate (*p*=0.0413) concentrations were elevated in the CKD group. The eGFR significantly decreased (*p*<0.0001) in the CKD group. Moreover, the urinalysis showed a reduction in urine creatinine level (*p*=0.0045), as well as a rise in urine protein concentration (*p*=0.0259) in the CKD group. Proteinuria was further supported by the substantial increase in the urine protein/creatinine ratio (*p*=0.0090).

**Table 3:** Results of the blood test and urinalysis 9 weeks after 5/6 nephrectomy. *p<0.05 vs sham; **p<0.01 vs sham; ***p<0.001 vs sham; *n*=6–11

	Sham		CKD		*p*
	Mean	SD	Mean	SD	
Serum Na^+^ (mmol/L)	141.9	2.424	142.5	2.659	0.5693
Serum K^+^ (mmol/L)	5.180	0.3190	5.736	0.7004	*0.0329
Serum Ca^2+^ (mmol/L)	2.601	0.05507	2.782	0.1414	**0.0013
Serum Mg^2+^ (mmol/L)	0.9490	0.1046	1.279	0.3390	**0.0083
Serum PO_4_^3-^ (mmol/L)	2.452	0.2227	2.873	0.5683	*0.0413
Serum Cl^-^ (mmol/L)	102.4	1.075	102.5	3.503	0.9629
Serum carbamide (BUN) (mmol/L)	7.744	0.5151	18.76	3.230	***<0.0001
Serum creatinine (μmol/L)	24.67	2.693	70.88	17.62	***<0.0001
Estimated glomerular filtration rate (eGFR) (μL/min)	3117	372.2	659.7	295	***<0.0001
Urine creatinine (μmol/L)	7668	3499	3972	1668	**0.0045
Urine protein (mg/dL)	152.9	97.77	655.1	472.2	*0.0259
Urine protein/creatinine ratio (mg/g)	3185.8	2779.8	24826.6	16915.8	**0.0090

### Trp metabolism shifted from the indole and serotonin pathways to the kynurenine pathway

The results of plasma metabolite analysis are summarized in Table 4. Compared to the sham-operated group, in the CKD group a significant increase was found in the concentrations of pCS (*p*=0.0082), IS (*p*=0.0044), KYN (*p*=0.0190), KYNA (*p*=0.0045), 3-OH-KYN (*p*=0.0024), anthranilic acid (AA) (*p*=0.0096), xanthurenic acid (XA) (*p*=0.0199), 5-hydroxy indole acetic acid (5-HIAA) (*p*=0.0056), picolinic acid (PA) (*p*=0.0167), and quinolinic acid (QA) (*p*=0.0058). There was a significant decrease, however, in the levels of Trp (*p*=0.0009), tryptamine (TPA) (*p*=0.0206), 5-hydroxytryptophan (5-HTP) (*p*=0.0145), serotonin (5-HT) (*p*=0.0422), and tyrosine (Tyr) (*p*=0.0124). Both the KYN/Trp and 3-OH-KYN/KYN ratios increased in the CKD group, reflecting the activity of IDO/TDO and kynurenine 3-monooxygenase (KMO), respectively.

**Table 4:** Plasma metabolites measured by UHPLC-MS/MS 9 weeks after 5/6 nephrectomy. *p<0.05 vs sham; **p<0.01 vs sham; ***p<0.001 vs sham; *n*=7–8

	nmol/L	Sham		CKD		*t*(df)	*p*
		Mean	SD	Mean	SD		
Uremic toxins	**p-cresyl sulfate**	1937	1113	7876	4122	*t*(6.766)=3.696	**0.0082
	**Indoxyl sulfate**	3366	720.7	9319	3602	*t*(6.421)=4.298	**0.0044
HPA axis	**Corticosterone**	1206	289.5	1144	291.0	*t*(12.72)=0.4161	0.6843
	**Tryptophan**	67350	8067	50411	7189	*t*(12.99)=4.300	***0.0009
Kynurenine pathway	**Kynurenine**	1943	452.3	3464	1275	*t*(7.318)=2.995	*0.0191
	**Kynurenine/tryptophan**	0.0294	0.0083	0.0708	0.0282	*t*(6.918)=3.741	**0.0074
	**Kynurenic acid**	90.34	30.32	210.1	74.85	*t*(7.710)=3.958	**0.0045
	**Kynurenic acid/kynurenine**	0.0461	0.0081	0.0614	0.0175	*t*(8.247)=2.120	0.0658
	**3-OH-kynurenine**	21.00	5.882	68.03	25.47	*t*(6.561)=4.775	**0.0024
	**3-OH-kynurenine/kynurenine**	0.0108	0.0011	0.0202	0.0063	*t*(6.295)=3.938	**0.0069
	**Anthranilic acid**	14.40	4.669	26.33	8.592	*t*(8.987)=3.275	**0.0096
	**Xanthurenic acid**	77.65	29.13	153.9	64.3	*t*(8.124)=2.888	*0.0199
	**Quinaldic acid**	54.55	28.75	43.12	21.95	*t*(12.80)=0.8708	0.3999
	**Picolinic acid**	147.7	38.74	226.7	62.78	*t*(9.737)=2.883	*0.0167
	**Quinolinic acid**	269.7	178.4	1256	638.8	*t*(6.820)=3.953	**0.0058
Serotonin/indole pathway	**Tryptamine**	4.038	1.541	2.357	0.7713	*t*(10.57)=2.719	*0.0206
	**Indole-3-acetic acid**	684.1	273.2	697.8	341.7	*t*(11.50)=0.08492	0.9338
	**5-OH-tryptophan**	4.925	0.9910	3.608	0.8187	*t*(12.97)=2.818	*0.0145
	**Serotonin**	746.6	461.1	297.1	296.6	*t*(12.04)=2.272	*0.0422
	**5-hidroxy-indole-acetic acid**	86.49	15.31	210.7	79.12	*t*(6.394)=4.087	**0.0056
Tyrosine metabolites	**Tyrosine**	63070	6016	53384	6697	*t*(12.24)=2.930	*0.0124
	**3-O-methyldopa**	68.04	14.68	62.07	5.060	*t*(8.840)=1.079	0.3090

### Following 5/6 nephrectomy, the animals spent more time in the arms of the EPM, but the number of entries decreased

**Fig. 2:**
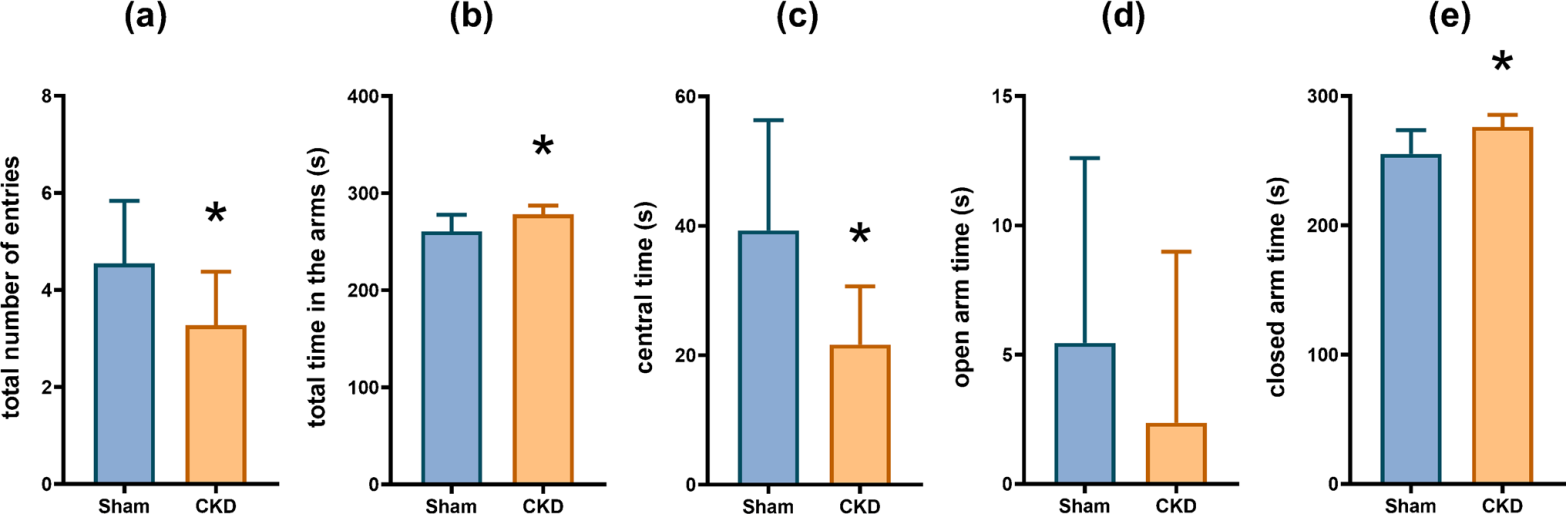
EPM test results: **a** total number of entries into the arms; **b** total time spent in the arms of the maze; **c** time spent in the center of the maze; **d** time spent in the open arms; **e** time spent in the closed arms; mean+SD, *n*=11; *p<0.05 vs sham

In the EPM test, the Mann-Whitney test revealed a significant reduction in the total number of entries into the arms (Fig. 2a; Mdn_SHAM_=5, Mdn_CKD_=3, *U*=26.5, *p*=0.0182), but the nephrectomized rats still spent more time in the arms of the maze (Fig. 2b; Mdn_SHAM_=263, Mdn_CKD_=276, *U*=28, *p*=0.0344) and less time in the center (Fig. 2c; Mdn_SHAM_=37, Mdn_CKD_=24, *U*=28, *p*=0.0344) than the sham-operated group. Considering the time spent in each arm, there was a significant increase in the closed arm time (Fig. 2e, Mdn_SHAM_=259, Mdn_CKD_=274, *U*=23.5, *p*=0.0135) and no significant difference was detected in the open arm time (Fig. 2d, Mdn_SHAM_=0, Mdn_CKD_=0, *U*=44, *p*=0.2337). There was no significant difference in the number of entries into the open arms (Mdn_SHAM_=0, Mdn_CKD_=0, *U*=45.5, *p*=0.3473) or the closed arms (Mdn_SHAM_=4, Mdn_CKD_=3, *U*=33, *p*=0.0713).

### Following 5/6 nephrectomy, central locomotion, and rearing decreased in the OF test

**Fig. 3:**
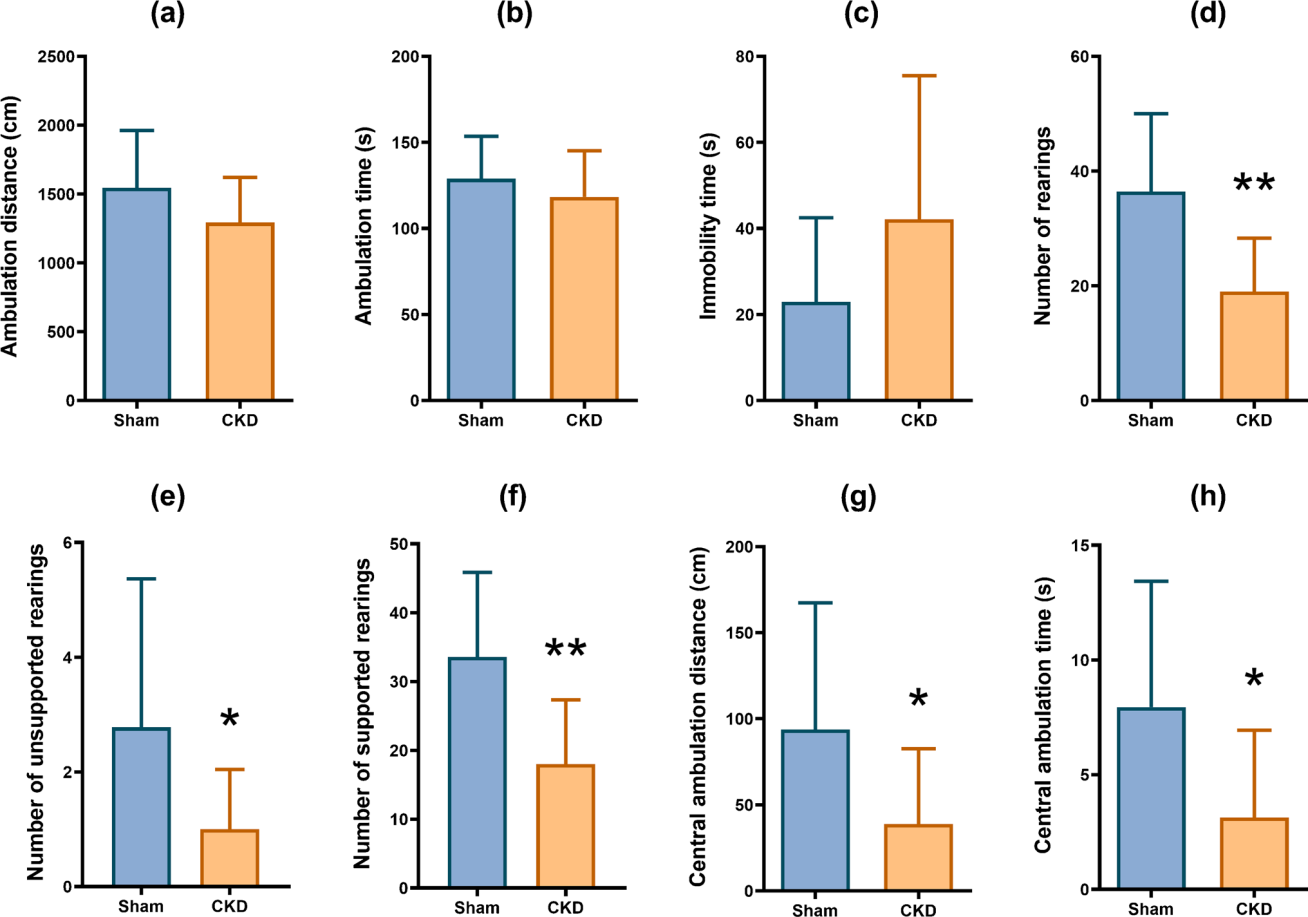
Computerized OF test results: **a** total distance covered, **b** total time of ambulation, **c** total time spent immobile, **d** number of rearings; mean+SD; **e** number of unsupported rearings, **f** number of supported rearings, **g** distance covered in the central zone, **h** time spent in the central zone; *n*=9–12; *p<0.05 vs sham; **p<0.01 vs sham

In the OF test, neither the total distance covered in the OF arena (Fig. 3a; *M*_SHAM_ =1544, *M*_CKD_=1293, *t*(19)=1.549, *p*=0.1380) nor the time spent with locomotion (Fig. 3b; *M*_SHAM_ =128.9, *M*_CKD_ =118.3, *t*(19)=0.9236, *p*=0.3673) was significantly affected by the induction of CKD. Likewise, no significant difference could be seen in the time spent immobile (Fig. 3c; *M*_SHAM_ =22.94, *M*_CKD_ =42.13, *t*(19)=1.532, *p*=0.1419). There was a significant reduction in the number of rearings in the CKD group (Fig. 3d; *M*_SHAM_ =36.44, *M*_CKD_ =19.00, *t*(19)=3.502, *p*=0.0024), which affected both the unsupported (Fig. 3e; *M*_SHAM_ =2.778, *M*_CKD_ =1.00, *t*(19)=2.170, *p*=0.0429) and the supported rearing activity (Fig. 3f; *M*_SHAM_ =33.56, *M*_CKD_ =18.00, *t*(19)=3.301, *p*=0.0038). Moreover, the nephrectomized group covered a significantly shorter distance in the central zone of the arena compared to the sham-operated animals (Fig. 3g; *M*_SHAM_ =93.78, *M*_CKD_ =38.85, *t*(19)=2.139, *p*=0.0456), and they also spent less time in the central zone (Fig. 3h; *M*_SHAM_ =7.944, *M*_CKD_ =3.125, *t*(19)=2.380, *p*=0.0280).

### The number and time of interactions with marbles in the MB test decreased in CKD

**Fig. 4:**
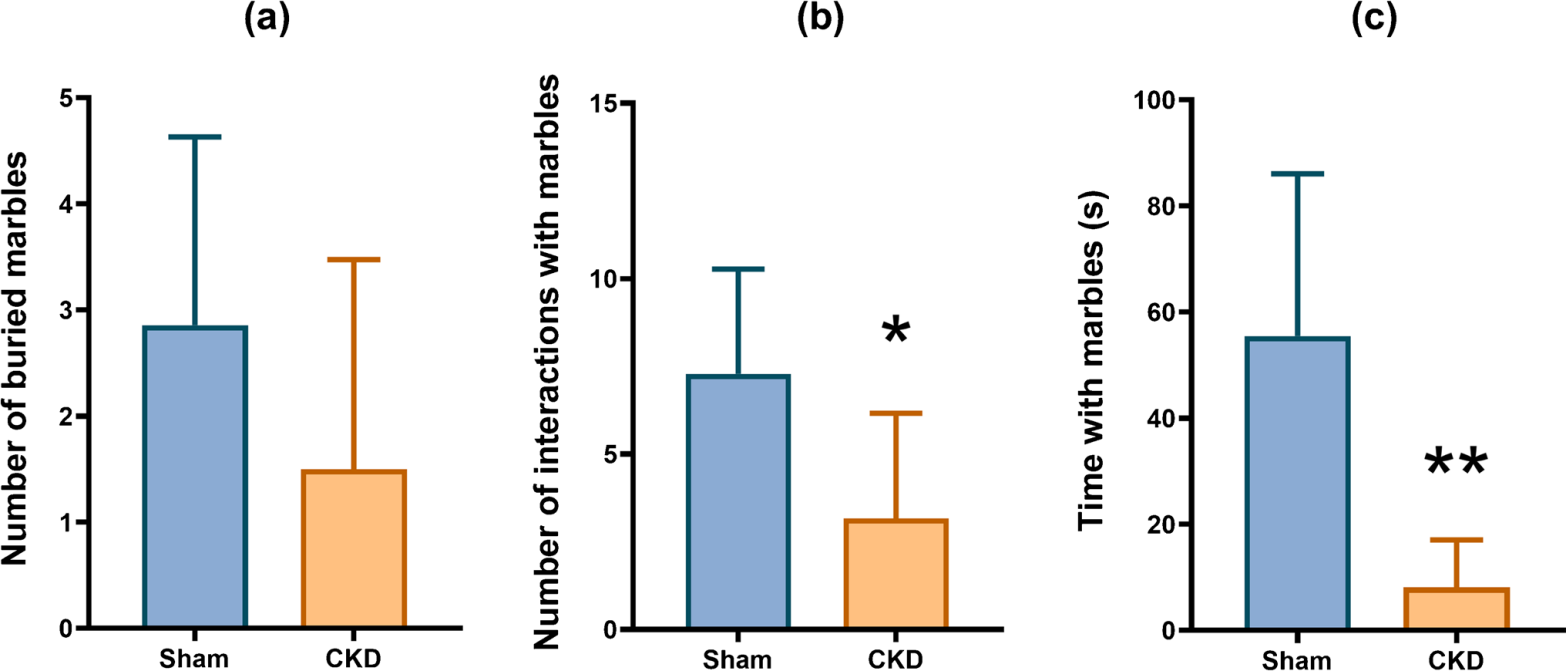
MB test results **a** number of buried marbles, **b** number of interactions with marbles, **c** time spent with marbles; mean+SEM; *n*=6–7; *p<0.05 vs sham

In the MB test, Welch’s *t*-test could not detect any significant difference in the number of buried marbles (>50% covered with bedding material) between the groups (Fig. 4a, *M*_SHAM_ =2.857, *M*_CKD_ =1.5, *t*(10.23)=1.295, *p*=0.2239). Interestingly, the CKD group interacted with the marbles fewer times (Fig. 4b, *M*_SHAM_ =7.286, *M*_CKD_ =3.167, *t*(10.68)=2.476, *p*=0.0313) and for a shorter amount of time (Fig. 4c, *M*_SHAM_ =55.5, *M*_CKD_ =8.115, *t*(7.168)=3.914, *p*=0.0055) than the sham-operated rats.

### *Crh*, *Crhr1*, and *Crhr2* were upregulated in the amygdala following 5/6 nephrectomy

**Fig. 5:**
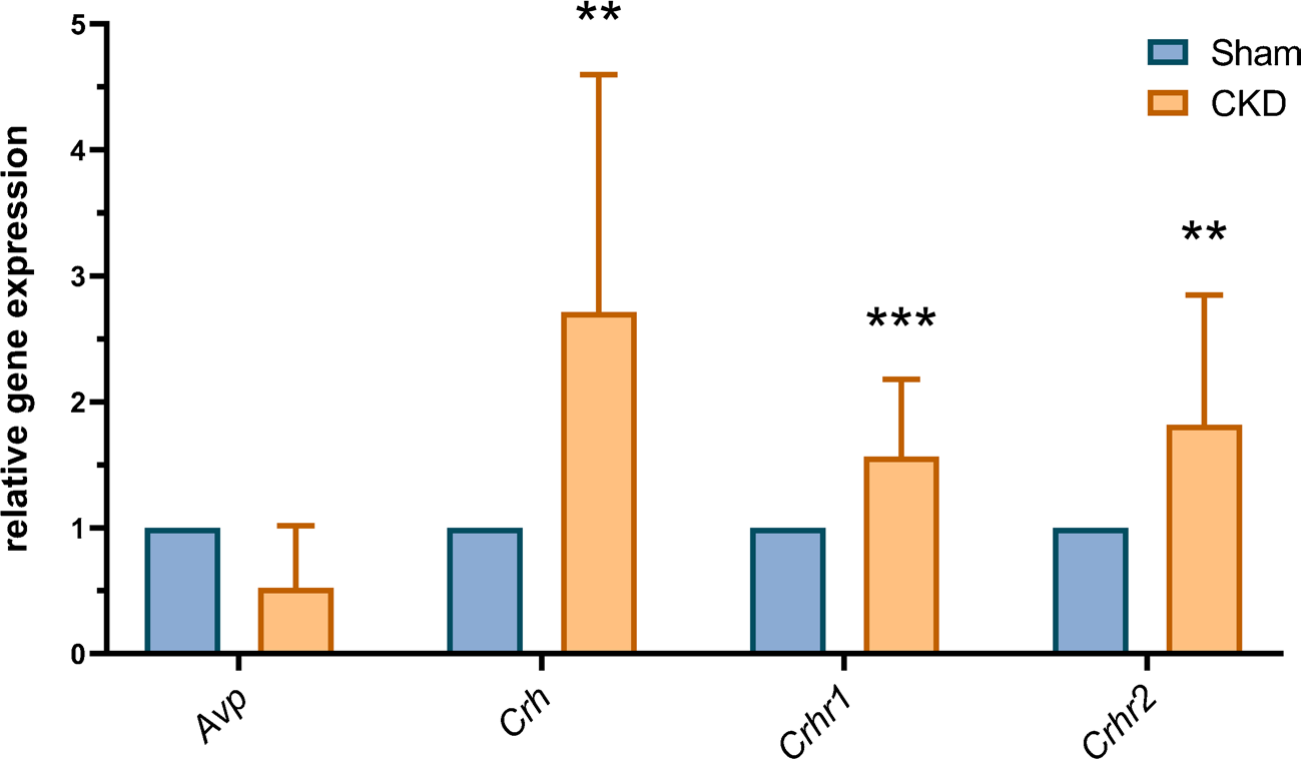
Relative gene expression in the amygdala; mean+SD; *n*=5–8; **p<0.01 vs sham; ***p<0.001 vs sham

The expression of each gene was calculated compared to *Gapdh* expression and analyzed using the Mann-Whitney test. As seen in Fig. 5, the mRNA expression of *Crh* (Mdn_SHAM_=1, Mdn_CKD_=2.065, *U*=7, *p*=0.0090), *Crhr1* (Mdn_SHAM_=1, Mdn_CKD_=1.390, *U*=0, *p*=0.0002), and *Crhr2* (Mdn_SHAM_=1, Mdn_CKD_=1.429, *U*=0, *p*=0.0079) in the amygdala was significantly higher in the CKD group than in the sham-operated group, whereas *Avp* expression (Mdn_SHAM_=1, Mdn_CKD_=0.47, *U*=16, *p*=0.1462) was not affected significantly by the 5/6 nephrectomy.

### There was no significant alteration in protein expression in the amygdala

**Fig. 6:**
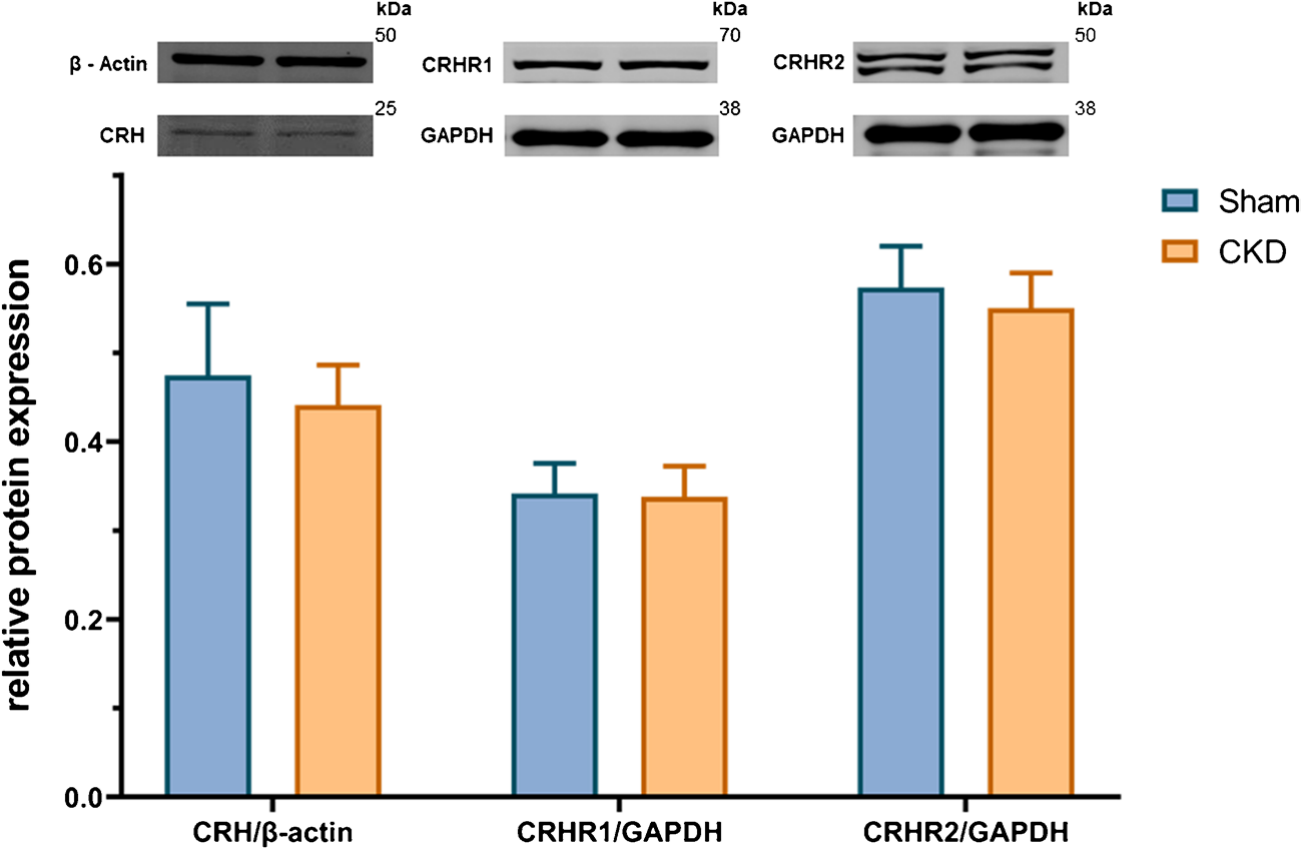
Protein expression in the amygdala; mean+SD; *n*=11–12

In the amygdala (Fig. 6), no significant difference was found in the protein expression of CRH (*M*_SHAM_ =0.4747, *M*_CKD_ =0.4413, *t*(17.23)=1.251, *p*=0.2276), CRHR1 (*M*_SHAM_ =0.3417, *M*_CKD_ =0.3379, *t*(22)=0.2684, *p*=0.7909) and CRHR2 (*M*_SHAM_=0.5737, *M*_CKD_ =0.5503, *t*(17.23)=1.302, *p*=0.2070) between the sham-operated and CKD groups. The expression of CRH was determined using β-actin as a loading background, whereas GAPDH was employed for the analysis of CRHR1 and CRHR2 expression. Pictures of the original, uncropped gels are included in the Supplement (Figs. S2-4).

## Discussion

Subtotal (5/6) nephrectomy is a widely used, reliable model of CKD in rodents. It mimics the mechanism of CKD secondary to nephron loss in humans, characterized by progressive glomerulosclerosis and tubulointerstitial fibrosis [91]. Subtotal nephrectomy can be performed by either ligation of the polar branches of the renal artery (ligation model) or by excision of the poles (ablation model), followed by contralateral nephrectomy. Although the ligation model is associated with more severe hypertension and proteinuria [4], the extent of resulting nephron loss is fairly unpredictable, causing a significant variation in the severity of CKD. However, the ablation model seems to be more reproducible with satisfactory inter-individual variation [52]; thus, we chose this method for CKD induction.

To confirm the development of CKD, urine creatinine and protein levels, as well as serum carbamide, creatinine, lipid, and ion levels, were measured 8 and 9 weeks after nephrectomy, respectively. Furthermore, the eGFR and urine protein/creatinine ratio were calculated. The significant increase in serum carbamide and creatinine levels, as well as the decrease in eGFR confirm the deterioration of renal function in the nephrectomized animals. Additionally, we found that the urine protein/creatinine ratio increased in the CKD group, which indicates damage to the filtration barrier [90]. Based on the study of Ormrod and Miller, the average serum creatinine level of 19.98 μmol/L corresponds to a moderate level of uremia [61].

Results of the blood test and urine analysis underline the presence of CKD since we detected a significant elevation of serum potassium and phosphate as well as calcium and magnesium. Potassium and phosphate retention are well-known consequences of CKD [32, 87], whereas the elevation of serum calcium concentration could result from the development of secondary hyperparathyroidism. In fact, secondary hyperparathyroidism is known to develop from stage 2 CKD in humans, and a four-fold increase in parathyroid hormone (PTH) level can be observed when end-stage kidney disease develops [20]. Besides, the excretion of magnesium decreases with the advancement of CKD, typically causing hypermagnesemia from stage 4 [58]. Thus, the increase in magnesium concentration might be also due to the impaired renal excretion in the CKD group.

Considering the controversial results available in the literature on the effect of CKD on anxiety-related behavior, first, we conducted a battery of behavioral tests, known to be sensitive for detecting anxiety-related behaviors. The EPM test is probably the most frequently used behavioral test for the assessment of anxiety-like behavior in rodents [85]. It is an ethologically based, the so-called approach-avoidance test, based on the innate conflict between the rodents’ drive to explore the novel environment and their fear of exposed areas [11]. The time spent in the open arms and the number of entries into the open arms negatively correlate with anxiety-like behavior [21]. In our study, the CKD group spent less time in the central area and more time in the closed arms of the EPM than the sham-operated animals. The data on the ambulatory activity of the animals is somewhat contradictory since nephrectomy seems to induce a decrease in the total entries into arms, whereas it increases the total time spent in arms. Nevertheless, taking these results together with that of the OF experiments, we conclude that it is unlikely that 5/6 nephrectomy affects locomotor activity. Therefore, the increased closed-arm time indicates an avoidance of open arms, which is a sign of anxiety-like behavior [82]. It should be noted, however, that no significant difference was detected in the open-arm entries and open-arm time, so the results should be interpreted with caution.

Since its development in 1934 as a test for the assessment of emotionality in rodents, the OF test has become a widely used model in preclinical tests of anxiolytic drugs. In this ethologically based test, the anxious animals tend to exhibit wall-hugging behavior (thigmotaxis), avoiding the central part of the apparatus [64]. Nine weeks after the nephrectomy, a significant decrease in the time spent in the central zone and the distance covered in the central zone was observed in the CKD group, which can be interpreted as anxiety-like behavior. Furthermore, the CKD group exhibited a decrease in rearing behavior, affecting the number of supported and unsupported rearings equally. Rearing—i.e., temporarily standing on the hind legs—is generally considered an exploratory behavior [75]. However, its relation to anxiety is rather controversial, as some studies have associated increased anxiety with increased rearing [6, 30], while others have found an increase in rearing after treatment with anxiolytic drugs [18, 26]. Recent studies have suggested that only unsupported rears (without leaning to a wall) correlate with emotionality in rodents [75] and can be considered a hippocampus-related exploratory behavior [50]. In addition, an increased tendency in the immobility time was observed in the CKD group suggesting freezing behavior characteristic of anxiety. In our study, there was no change in ambulation time and ambulation distance, so the total ambulatory activity was not affected by the nephrectomy. Therefore, the reduction in rearing could more likely be attributed to the suppression of exploratory behavior, which is common in anxiety-provoking, aversive circumstances [75].

Overall, our results suggest that CKD indeed induces anxiety-like behavior. This is in accordance with the study of Chandanathil et al., who reported a decrease in peripheral square crossings and in the number of rearings both four and eight weeks after nephrectomy in rats [13]. Another group has also observed a lower number of square crossings in the OF test in moderate CKD, as well as a reduction in rearing activity in severe CKD, 1 month after nephrectomy [79]. In a recent study, anxiety-like behavior has been observed in the EPM, light-dark box, and tail suspension tests in CKD induced by an adenine-rich diet in mice [33].

In contrast with our observations, Tóthova et al. have not found any significant behavioral alteration in rats 3 months after 5/6 nephrectomy. Nine months after nephrectomy, however, the animals spent more time in the light compartment of the light-dark box, which suggests an anxiolytic-like effect, although the reduction of anxiety-like behavior was not confirmed by the OF test [80]. It should be noted, that most studies reported anxiety-like behavior 4-8 weeks after nephrectomy [13, 79], and in the study of Chandanathil et al., the reduction in exploratory behavior was no longer significant 12 and 16 weeks after nephrectomy [13]. In a murine model of CKD, anxiolytic-like behavior was reported 4 and 10 weeks after induction: the time spent in the light compartment of the light-dark box increased, whereas the closed arm time in the EPM test decreased. However, the reduction in closed-arm time could result from the robust increase in time spent at the central intersection of the arms, rather than from the avoidance of open arms [16]. This study has also employed a different CKD model (cortical electrocautery and nephrectomy) and a different species, which might account for some of these discrepancies.

To obtain a more comprehensive behavioral profile an MB test was also performed. To our knowledge, MB behavior has not yet been assessed in rodent CKD models. Burying behavior, i.e., the displacement of bedding material using the snout and forepaws in a concerted effort to cover a harmful or non-harmful object, is part of the rodents’ normal behavioral repertoire [8]. The MB test was originally devised to measure defensive burying as a sign of novelty-induced anxiety, but nowadays, it is frequently used as an assay of perseverative, compulsive-like behavior, as well [78]. Seven weeks after nephrectomy, no difference was found in the number of buried marbles between the groups. When considering the goal-oriented interactions with marbles, however, a significant reduction in their duration and number was observed in the CKD group. As the general locomotor activity did not seem to be affected in the OF and EPM, our MB results may correlate with a reduction in explorative behavior.

Considering our findings of an anxiety-inducing effect of 5/6 nephrectomy after 7 weeks, we aimed to investigate the possible mechanism of action via assessing the presence of uremic toxins associated with anxiety as well as analyzing HPA axis activity including amygdalar expression of key anxiogenic genes.

Uremic toxins might mediate anxiety-like behavior in our study, as they have been implicated in the neuropsychiatric complications of CKD by several groups. In CKD patients, serum indole-3-acetic acid concentration has correlated with anxiety, depression, and the quality of sleep [39]. In rodent models of CKD, both pCS [77] and IS have been linked to anxiety-like behavior [38, 76]. In fact, Karbowska et al. found that a high dose of IS (200 mg/kg) administered to intact rats caused an increase in stress sensitivity [38]. These data indicate that, indeed, uremic toxins can be in the background of CKD-induced anxiety.

IS has also reduced brain serotonin, dopamine, and norepinephrine levels in rats [38]. The robust increase in the concentration of both IS and pCS in our study detected at week 9 could cause the reduced exploration indirectly by decreasing the cerebral monoamine concentrations. In point of fact, fluvoxamine (a selective serotonin reuptake inhibitor) and bupropion (an atypical antidepressant) have increased exploration around the marbles in the MB test in mice [29]. Furthermore, these gut bacteria-derived, protein-bound uremic toxins (IS and pCS) have a well-established proinflammatory effect [83], which in turn has been implicated in the development of anxiety [19].

Recently, Huang et al. have also pointed to the involvement of high serum urea levels in the development of CKD-related anxiety. In their study, the severity of anxiety-related behavior has correlated with serum urea concentration in a mouse model of CKD. Moreover, urea transporter B KO mice have shown an increase in anxiety-like behavior, compared to WT mice, which could indicate the anxiogenic effect of urea per se [33]. In our study, the urea concentration has risen robustly in the CKD group; therefore, it could also contribute to the development of anxiety.

**Fig. 7:**
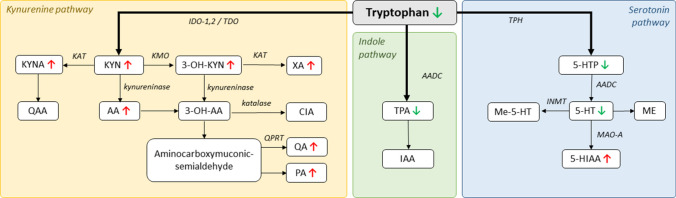
Overview of the alterations in the plasma concentration of tryptophan metabolites following 5/6 nephrectomy. The arrows indicate significant changes compared to the sham-operated group. 3-OH-AA: 3-hydroxyanthranilic acid; 3-OH-KYN: 3-hydroxykynurenine;5-HIAA: 5-hydroxyindole-3-acetic acid; 5-HT: serotonin; 5-HTP: 5-hydroxy-L-tryptophan; AA: anthranilic acid; CIA: cinnabaric acid; IAA: indoleacetic acid; IDO-1,2: indoleamine 2,3-dioxygenase; INMT: indolethylamine-N-methyltransferase; KAT: kynurenine aminotransferase; KMO: kynurenine 3-monooxygenase; KYN: kynurenine; KYNA: kynurenic acid; MAO-A: monoamine oxidase A; ME: melatonin; Me-5-HT: methylserotonin; PA: picolinic acid; QA: quinolinic acid; QAA: quinaldic acid; TDO: tryptophan 2,3-dioxygenase ; TPA: tryptamine; TPH: tryptophan hydroxylase; XA: xanthurenic acid

Furthermore, in our experiments, the metabolism of tryptophan has shifted from the indole and serotonin pathways to the kynurenine pathway (see Fig. 7). In CKD, multiple studies have reported an increase in KYN metabolites [7, 88], behind of which is most likely chronic inflammation, as inflammatory cytokines (such as IFN-γ, TNF-α) activate IDO, the rate-limiting enzyme of KYN synthesis [95]. KYN metabolites play a controversial role in the pathomechanism of CKD, as some of them seem to have a detrimental effect, whereas others might be considered protective [56]. For instance, 3-OH-KYN has been associated with mitochondrial dysfunction and increased ROS production [66], but it has been reported that KYNA has anti-inflammatory properties and it prevents homocysteine-induced damage to the endothelium [3, 89]. Our results showed an increase in the KYN/Trp ratio indicating an activation of IDO and/or TDO, which is in accordance with previous results [37, 62]. TDOs are mainly localized in the liver and can be activated in response to glucocorticoids. However, most of the kynurenine synthesis in inflammatory diseases is extrahepatic, as cytokines activate IDOs in the central nervous system, blood, spleen, kidneys, and lungs [42]. In our study, there was a robust increase in the 3-OH-KYN/KYN ratio, pointing to the activation of KMO. In the presence of KMO, neurotoxic and excitatory products of KYN are synthesized, including 3-OH-KYN and QA that have been associated with free radical production and excitotoxicity, respectively [15]. In comparison, we observed a less pronounced increase in KYNA synthesis. These results suggest that CKD induced an imbalance between the neuroprotective KYNA and the abovementioned neurotoxic products [34]. In fact, KYN and QA are not only neurotoxic but they have also been associated with anxiety in preclinical and clinical studies [48, 60], as opposed to the anxiolytic role of KYNA [47]. The predominance of anxiogenic mediators is further amplified by 5-HT deficiency due to the shift of Trp metabolism to the KYN pathway in inflammation, which further increases the sensitivity to anxiety [42].

Since HPA axis dysregulation has previously been implicated in CKD, we investigated on one hand if indeed the endocrine HPA axis activity is altered and on the other hand if the expression of CRH and its receptors is influenced by our CKD model.

As an indicator of the endocrine HPA axis, we measured the plasma corticosterone 8 weeks after CKD induction. Our results showed that in our model CKD did not alter plasma corticosterone concentration. These results are in line with the findings of Lu et al., in which plasma corticosterone has not changed in either male or female rats 7 weeks after 5/6 nephrectomy [53]. Another study has investigated plasma ACTH and corticosterone levels at multiple time points following 5/6 nephrectomy in rats, and the increase in corticosterone has only become significant 90 days after the nephrectomy [31]. Based on these data, it is possible that our sample collection happened too soon for a significant elevation in plasma corticosterone to develop.

CRH is expressed in a variety of anxiety-related extrahypothalamic sites of the central nervous system, including the central amygdala (CeA) [44]. In our gene expression analysis, significant upregulations of *Crh*, *Crhr1*, and *Crhr2* were observed in the amygdala in the CKD group, which is in accordance with the development of anxiety-like behavior. In a study by Cipriano et al., the intra-amygdalar administration of CRH resulted in a reduction in open arm time and entries in the EPM test, while a selective CRHR1 antagonist induced an anxiety-like effect, pointing to the involvement of amygdalar CRHR1 signaling in anxiety [17]. Moreover, the continuous overexpression of CRH by a lentiviral vector in the central nucleus of the amygdala has been associated with the dysregulation of the HPA axis, increased acoustic startle response, as well as depressive-like behavior [40]. *Crhr2* upregulation can also be linked to anxiety, as urocortin 2 (a CRHR2 agonist) induced anxiety-like behavior, when injected into the medial amygdala in rats [1].

Contrary to the gene expression results, no significant differences were detected in the amygdalar protein expression of CRH and its receptors. The difference between mRNA and protein expression results might be due to multiple reasons.

Firstly, it is possible that an increase in transcription might not manifest in increased translation. It is well known that gene expression is regulated at multiple levels: transcription, mRNA processing, transport, and degradation as well as protein translation and degradation. Therefore, protein levels vary on a high dynamic range [73]. The rate of translation is regulated by a multitude of molecular mechanisms, transcription is only one of them. Additionally, the activity of eukaryotic initiating factors (EIFs), structural features (e.g., internal ribosome-entry sequences, upstream open reading frames, and secondary or tertiary RNA structures), RNA-binding proteins, as well as microRNAs and small interfering RNAs are involved in the regulation of translation [25]. Any of these mechanisms might be responsible for our results; therefore, it is possible that the increased expression of the *Crh*, *Crhr1*, and *Crhr2* genes, that we detected in the amygdala, does not reflect in their protein concentration.

Secondly, it is also possible that the HPA axis dysregulation has not yet developed in our model, or it just started to develop at the time of sample collection, and later a significant change could have been detected. Indeed, the corticosterone concentration was not elevated either at the time of sample collection, supporting the idea that HPA axis dysregulation has not developed yet. In fact, Hirotsu et al. found that ACTH and corticosterone levels fluctuate during the course of CKD. Corticosterone showed an elevation after 90 days of CKD induction, then dramatically decreased and started to rise again after 150 days [31]. It is possible that during the course of CKD, anxiety develops as a result of an interplay between multiple CKD-induced adverse effects (e.g., uremic toxins [38, 77], urea [33], oxidative stress [36], HPA axis dysregulation [31]) that at different time points contribute to anxiety to varying degree.

Overall, we can conclude that the altered CRH signaling is probably not involved in the development of CKD-induced anxiety at the time when sample collection was done in our study. Nevertheless, further studies are needed to establish the exact role of the HPA axis in anxiety during the course of CKD.

## Conclusion

Based on our results 5/6 nephrectomy, indeed evokes anxiety-like behavior 7 weeks after surgery in Wistar rats. 5/6 nephrectomy induced moderate CKD suggested by the laboratory and urinalysis results. In our study, IS and pCS levels have risen in the CKD group, both of which have been previously implicated in anxiety. 5/6 nephrectomy caused a shift in tryptophan metabolism toward the kynurenine pathway, consequently accumulating anxiogenic and neurotoxic metabolites, such as KYN and QA. We also investigated if the dysregulation of the HPA axis might play a role in anxiety-like behavior in CKD. Our results showed no significant elevation in corticosterone concentration, suggesting that the HPA axis is not involved in anxiety in our CKD model, at least not at this time point. Still, some changes in gene expression were detected in the CRH system in the amygdala, but these were not detectable on the protein level. Based on these findings, the anxiety-like behavior at this time point in CKD progression is more likely to be mediated by uremic toxins and the shift in tryptophan metabolism than the dysregulation of the HPA axis. It is noteworthy to mention that other mechanisms (such as inflammation and oxidative stress) might also contribute; however, these were not explored in our study.

### Supplementary information

ESM 1 (PDF 1188 kb)

## Data Availability

Datasets generated and analyzed, and the supplemental material for this study are available in the Mendeley Data repository at doi: 10.17632/gg2nrgfnzb.1; https://data.mendeley.com/datasets/gg2nrgfnzb/draft?a=91893794-72f9-4804-8f73-a80c5a429c90.

## Declarations

This investigation conformed to the EU Directive 2010/63/EU. The animals were kept and handled during the experiments in accordance with the instructions of the University of Szeged Ethical Committee for the Protection of Animals in Research, which approved these experiments (XV./799/2019).
