# Supplementary material for: Chronic kidney disease may evoke anxiety by altering CRH expression in the amygdala and tryptophan metabolism in rats
Source: Pflugers Arch. 2023 Nov 22;476(2):179–96. doi: 10.1007/s00424-023-02884-y (PMC10791708; doi:10.1007/s00424-023-02884-y)
Supplement: Supplementary file 1 — (PDF 1188 kb) [file 424_2023_2884_MOESM1_ESM.pdf]

# Supplement

## Chronic kidney disease may evoke anxiety by altering CRH expression in the amygdala and tryptophan metabolism in rats

### *Pflügers Archiv - European Journal of Physiology*

Katalin Eszter Ibos[a*], Éva Bodnár[a], Hoa Dinh[b], Merse Kiss[c], Fanni Márványkövi[c], Zsuzsanna Z. A. Kovács[c], Andrea Siska[d], Imre Földesi[d], Zsolt Galla[e], Péter Monostori[e], István Szatmári[f], Péter Simon[f], Márta Sárközy[c#], Krisztina Csabafi[a#]

[a] Department of Pathophysiology, Albert Szent-Györgyi Medical School, University of Szeged, 1 Semmelweis utca, H-6725 Szeged, Hungary
[b] Department of Biochemistry, Bach Mai Hospital, 78 Giai Phong Street, Phuong Mai, Dong Da, Hanoi, 100000, Vietnam
[c] Department of Biochemistry and Interdisciplinary Centre of Excellence, Albert Szent-Györgyi Medical School, 9 Dóm tér, University of Szeged, H-6720 Szeged, Hungary
[d] Department of Laboratory Medicine, Albert Szent-Györgyi Medical School, University of Szeged, 6 Semmelweis utca, H-6725 Szeged, Hungary
[e] Metabolic and Newborn Screening Laboratory, Department of Pediatrics, Albert Szent-Györgyi Medical School, University of Szeged, 35-36 Temesvári körút, H-6726 Szeged, Hungary
[f] Institute of Pharmaceutical Chemistry and Stereochemistry Research Group, Eötvös Loránd Research Network, University of Szeged, 9 Zrínyi utca, H-6720 Szeged, Hungary.
*Correspondence: ibos.katalin.eszter@med.u-szeged.hu

**Contents:**

- S1: $^{1}$H-NMR spectrum of p-cresyl-sulphate
- S2: Western blot; uncropped, duplicate gels of CRH
- S3: Western blot; uncropped, duplicate gels of CRHR1
- S4: Western blot; uncropped, duplicate gels of CRHR2

**Figure S1**

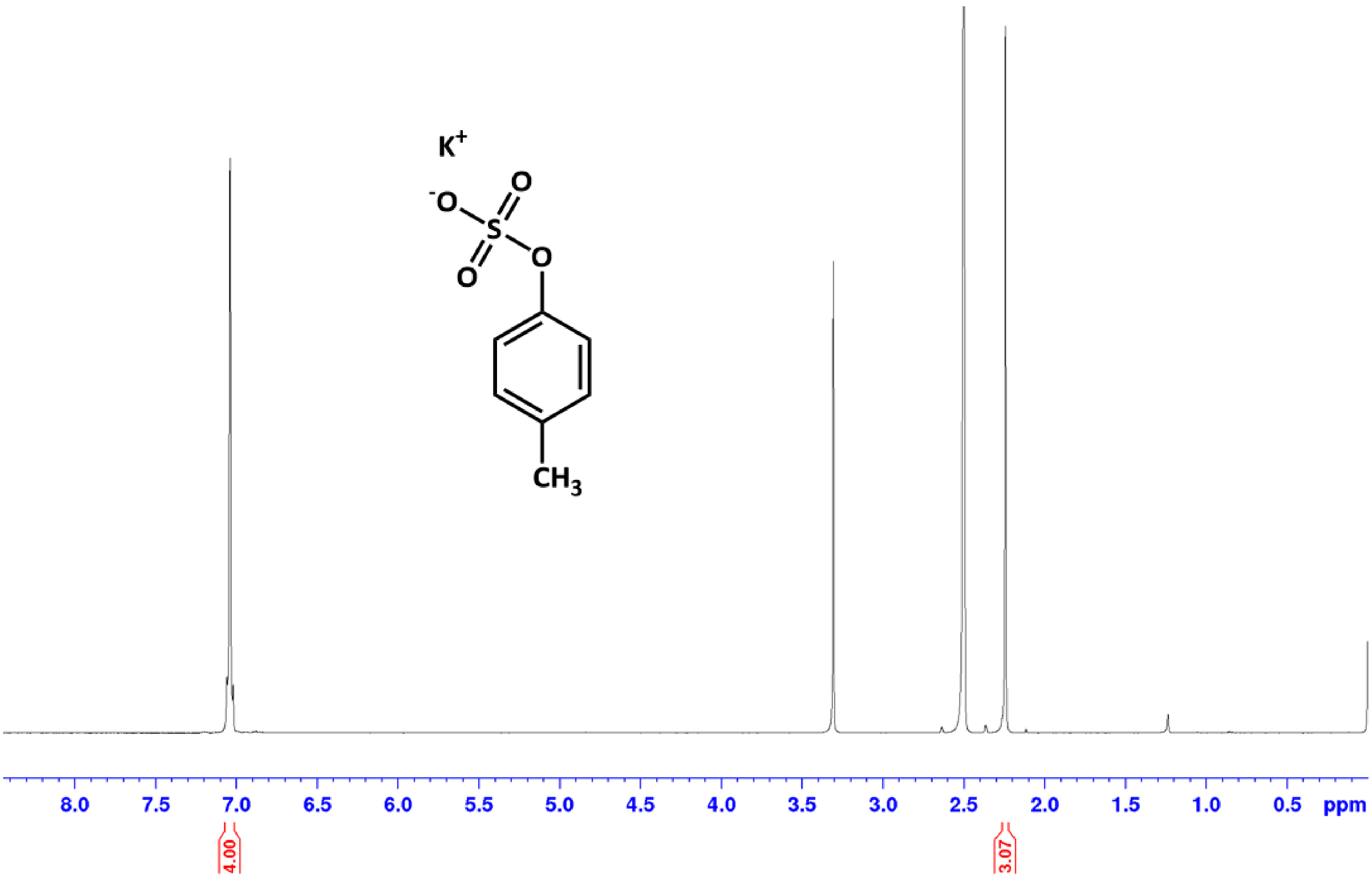
K+
-O
O
S
O
O
CH3
8.0
7.5
7.0
6.5
6.0
5.5
5.0
4.5
4.0
3.5
3.0
2.5
2.0
1.5
1.0
0.5
ppm
4.00
3.07

**Figure S2**

(a)

(b)

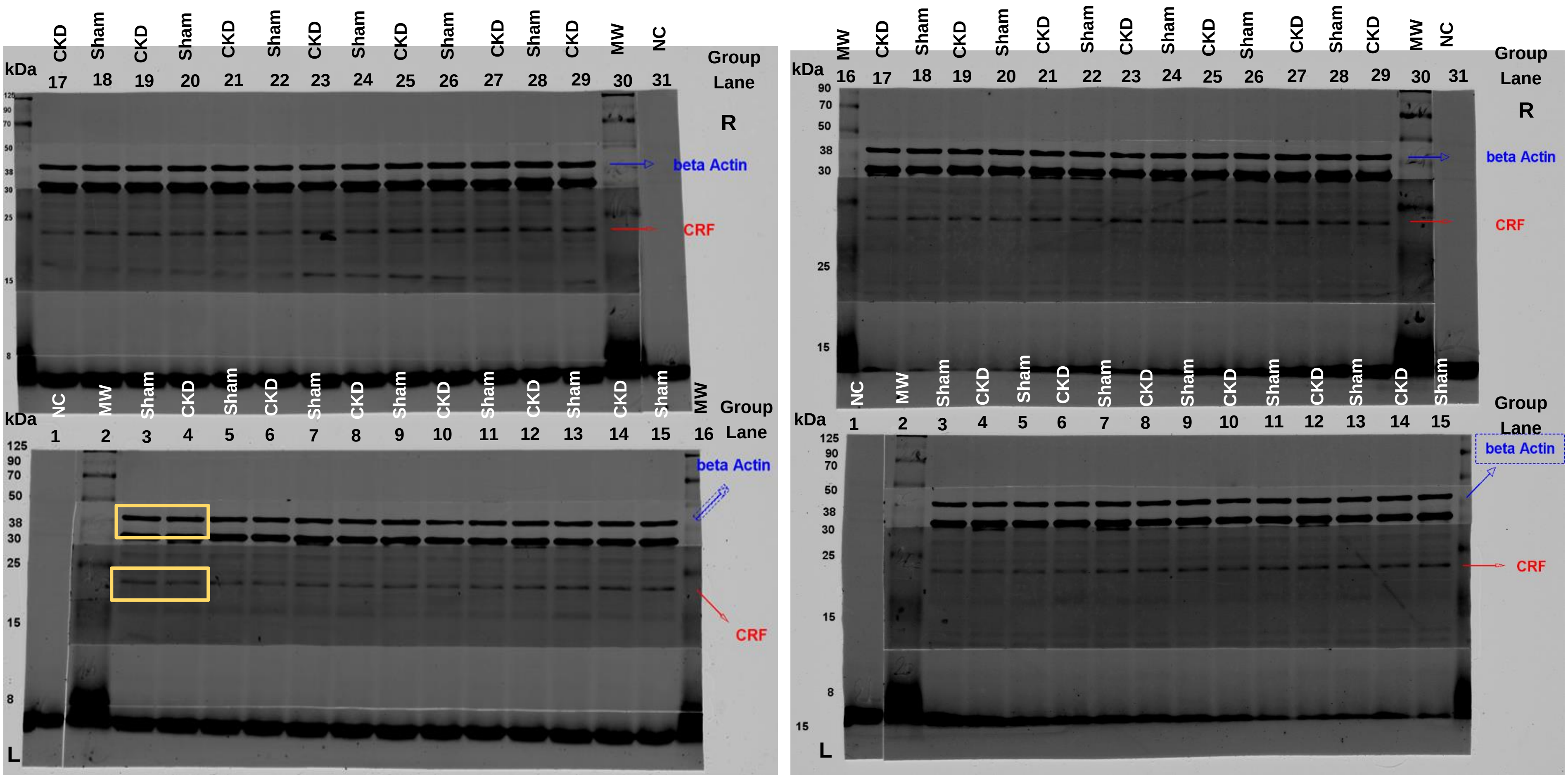

kDa
Group
Lane
CKD
Sham
MW
NC
R
L
beta Actin
CRF
125
90
70
50
38
30
25
15
8

## Figure S3

(a)

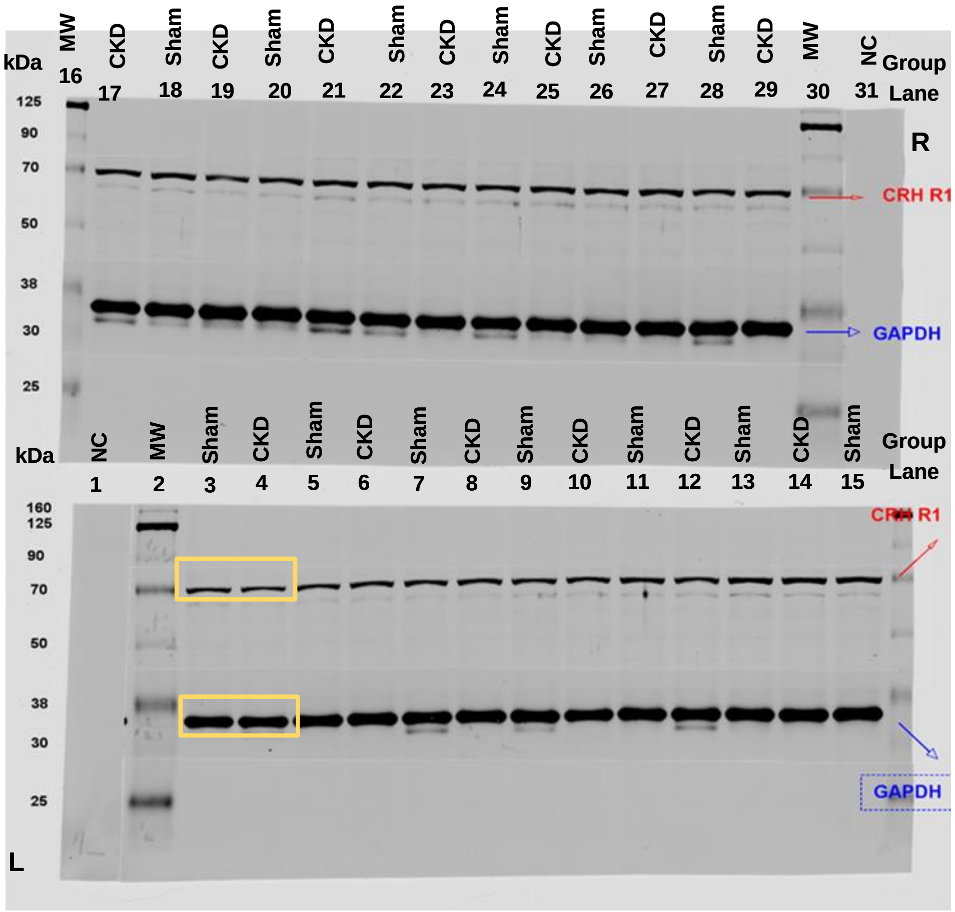
kDa
MW CKD Sham CKD Sham CKD Sham CKD Sham CKD Sham CKD Sham CKD MW NC Group
16 17 18 19 20 21 22 23 24 25 26 27 28 29 30 31 Lane
125
90
70
50
38
30
25
R
CRH R1
GAPDH
kDa
NC MW Sham CKD Sham CKD Sham CKD Sham CKD Sham CKD Sham CKD Sham Group
1 2 3 4 5 6 7 8 9 10 11 12 13 14 15 Lane
160
125
90
70
50
38
30
25
CRH R1
GAPDH
L

(b)

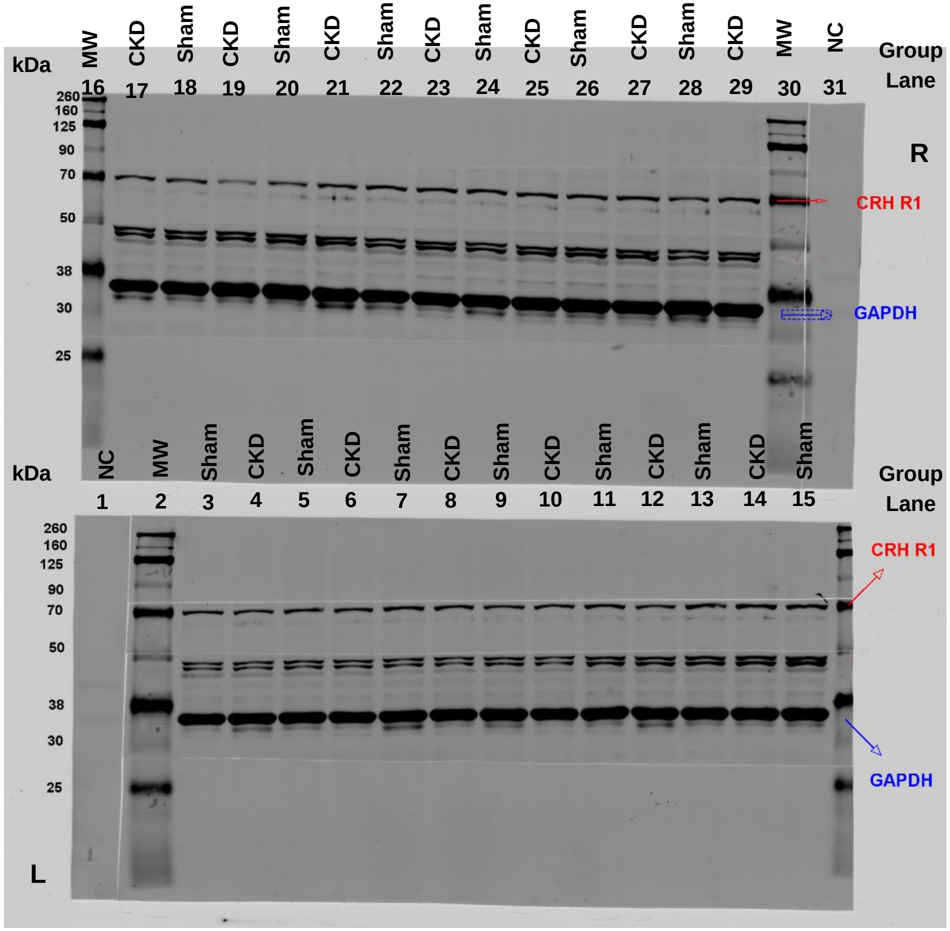
kDa
MW CKD Sham CKD Sham CKD Sham CKD Sham CKD Sham CKD Sham CKD MW NC Group
16 17 18 19 20 21 22 23 24 25 26 27 28 29 30 31 Lane
260
160
125
90
70
50
38
30
25
R
CRH R1
GAPDH
kDa
NC MW Sham CKD Sham CKD Sham CKD Sham CKD Sham CKD Sham CKD Sham Group
1 2 3 4 5 6 7 8 9 10 11 12 13 14 15 Lane
260
160
125
90
70
50
38
30
25
CRH R1
GAPDH
L

**Figure S4**

(a) (b)

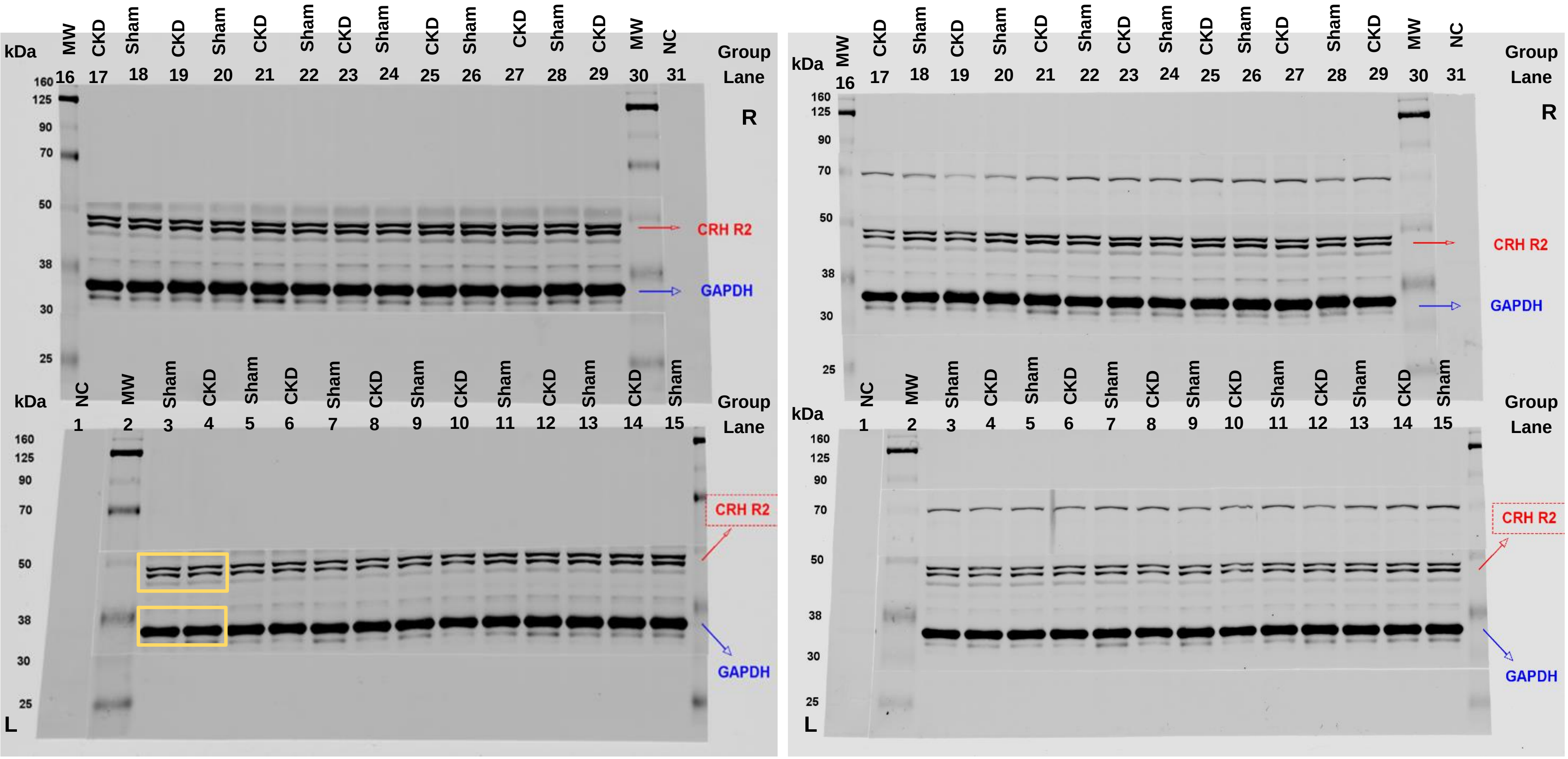
kDa
Group
Lane
MW CKD Sham CKD Sham CKD Sham CKD Sham CKD Sham CKD Sham CKD MW NC
16 17 18 19 20 21 22 23 24 25 26 27 28 29 30 31
160
125
90
70
50
38
30
25
R
CRH R2
GAPDH
NC MW Sham CKD Sham CKD Sham CKD Sham CKD Sham CKD Sham CKD Sham
1 2 3 4 5 6 7 8 9 10 11 12 13 14 15
L
